# Schoolyard physical activity of 6–11 year old children assessed by GPS and accelerometry

**DOI:** 10.1186/1479-5868-10-97

**Published:** 2013-08-14

**Authors:** Dirk Dessing, Frank H Pierik, Reinier P Sterkenburg, Paula van Dommelen, Jolanda Maas, Sanne I de Vries

**Affiliations:** 1 TNO, Department of Urban Environment and Safety, P.O. Box 80015, 3508 TA Utrecht, The Netherlands; 2 TNO, Lifestyle, P.O. Box 2215, 2301 CE Leiden, The Netherlands; 3 VU Medical Center, Department of Public and Occupational Health, EMGO Institute, P.O Box 7057 1007 MB Amsterdam, The Netherlands

**Keywords:** Physical activity, Schoolyard, Primary school, Children, Accelerometer, Global Positioning System (GPS)

## Abstract

**Background:**

Children’s current physical activity levels are disturbingly low when compared to recommended levels. This may be changed by intervening in the school environment. However, at present, it is unclear to what extent schoolyard physical activity contributes towards reaching the daily physical activity guideline. The aim of this study was to examine how long and at what intensity children are physically active at the schoolyard during different time segments of the day. Moreover, the contribution of schoolyard physical activity towards achieving the recommended guideline for daily physical activity was investigated.

**Methods:**

Children (n=76) between the age of 6–11 years were recruited in six different schools in five cities (>70.000 residents) in the Netherlands. During the weekdays of a regular school week, childrens’ physical activity and location were measured with ActiGraph accelerometers and Travelrecorder GPS receivers. Data was collected from December 2008 to April 2009. From the data, the amount of moderate to vigorous physical activity (MVPA) on and outside the schoolyard was established. Moreover, the percentage of MVPA on the schoolyard was compared between the following segments of the day: pre-school, school, school recess, lunch break and post-school. Differences between boys and girls were compared using linear and logistic mixed-effects models.

**Results:**

On average, children spent 40.1 minutes/day on the schoolyard. During this time, boys were more active on the schoolyard, with 27.3% of their time spent as MVPA compared to 16.7% among girls (OR=2.11 [95% CI 1.54 - 2.90]). The children were most active on the schoolyard during school recess, during which boys recorded 39.5% and girls recorded 23.4% of the time as MVPA (OR=2.55 [95% CI: 1.69 - 3.85]). Although children were only present at the schoolyard for 6.1% of the total reported time, this time contributed towards 17.5% and 16.8% of boys’ and girls’ minutes of MVPA.

**Conclusions:**

On the schoolyard, children’s physical activity levels are higher than on average over the whole day. Physical activity levels are particularly high during school recess. The school environment seems to be an important setting for improving children’s physical activity levels. Further research on the facilitators of these high activity levels may provide targets for further promotion of physical activity among children.

## Background

Physical activity is an important lifestyle factor that is associated with a wide range of health benefits [1-3]. When compared to recommended levels, children’s current physical activity levels are disturbingly low [4,5]. Dutch Standards for Healthy Activity [6] and recommendations by the WHO [7] state that children should participate in at least 60 minutes of moderate to vigorous physical activity (MVPA) every day. Recent data suggest that only 30% to 40% of children meet this requirement [4,5]. Since these low levels of physical activity in childhood are likely to continue into adulthood and can have a significant impact on future public health [8,9], it is clear that young children should be encouraged to be more physically active.

In the past decades, the ecological approach towards increasing physical activity has generated much interest among researchers and interventionists. Focus of research has shifted more and more towards the children’s environment [10]. This has led to growing evidence that the built environment is associated with children’s levels of physical activity [11-13]. Since young children spend a considerable part of their day in the school environment (for this paper defined as: inside the school and on the schoolyard) it is considered an especially important setting for promoting children’s physical activity [14-16].

Because nearly all children attend to school, the schoolyard provides the opportunity to reach almost all children. Several studies have already described children’s physical activity during school recess using the objective method of accelerometers [17-19]. These studies indicate that school recess is an important context for children’s physical activity. However, the schoolyard also provides opportunities for physical activity on other moments of the day (i.e. before and after school). No previous study has described the physical activity levels on the schoolyard during and outside school hours. Although the physical activity levels at the schoolyard are expected to be relatively high, it is unclear to what extent it contributes to achieving the standards for healthy physical exercise. Insight in to the contribution of specific locations (e.g. the schoolyard) to the accumulation of healthy physical activities is relevant for future recommendations to promote an healthy active lifestyle.

To be able to investigate how much time of their day children actually spend on the schoolyard during and outside school hours, exact information on the location of the children is essential. Whereas location was traditionally recorded by self-report or observations, the availability of small and accurate GPS devices has opened a new venue for more practical and objective personal tracking [20]. The present study combines positional data from GPS receivers with data from accelerometers. The additional GPS information on children’s location enables the assessment of the contribution of total physical activity on the schoolyard towards children’s total daily physical activity. It is of interest to study the differences in physical activity levels on different locations and times of the day for boys and girls, in order gain insight whether different interventions are needed by gender.

The aim of this study was to examine how long and at what intensity boys and girls are physically active at the schoolyard during different time segments of the whole day. Moreover, the contribution of schoolyard physical activity towards achieving the daily MPVA requirements was investigated.

## Methods

### Participants and setting

The present study was part of the Spatial Planning and Children’s Exercise (SPACE) study, which examined the relationship between the built environment and physical activity among school-aged children. The SPACE study was conducted in five neighborhoods that were due to be (partially) restructured between 2004 and 2008. These neighborhoods were located in five different municipalities with >70.000 residents in the Netherlands, i.e., Amersfoort, Haarlem, Hengelo, Rotterdam and Vlaardingen. In all neighborhoods, physical activity levels of primary school children of twenty schools were monitored through a 7-day physical activity diary, first in 2004 (n=401) and then once more in 2008 (n=292). Moreover, built environmental characteristics were collected through neighborhood observation. For further information, also see de Vries et al. [11] and de Vries et al. [21].

In 2008, from the twenty schools involved in the SPACE study, a convenience sample of six primary schools also agreed to participate in the present study. The neighborhoods of these six schools were similar in type of buildings (i.e., residence type, year of construction) and demographics (i.e., age distribution, social economic status, ethnicity). Moreover, the school building and the schoolyard of the six schools were comparable in size, with most of the schoolyard area that consisted of paved surface. All schools had only one schoolyard and were comparable in available fixed and portable equipment on the schoolyard.

Children that attended the six participating schools were invited through letters and pamphlets that were handed out by their teachers, resulting in a group of 97 children that were asked to wear accelerometers and GPS receivers. Informed consent was obtained from a parent or guardian of all participating children. Data were collected for one week per subject, in the period between December 2008 and April 2009. Average daytime temperatures were collected through a database of the *Royal Netherlands Meteorological Institute.* During data collection, average daytime temperatures ranged from 1 to 6 degrees Celsius. The study was approved by the ethics committee (IRB) of the Leiden University Medical Center.

### Instrumentation/measures

Physical activity was measured every 15 seconds with an uniaxial accelerometer (GT1M, ActiGraph, Pensacola, Florida). Longer (e.g. 1 minute) sampling intervals might have masked short intermittent bursts of physical activity that are typical for young children [22]. In addition to an accelerometer, children simultaneously carried a GPS receiver (Travel recorder X, BT-Q1000X, QStarz International Co) which recorded the geographical location every 5 seconds (positional accuracy of <3 m Circular Error Probability CEP (50%). Both recording devices are so-called ‘black boxes’. Meaning that during recording, children could not see anything indicating the measurements by the device.

Accelerometers and GPS receivers were distributed among the children during school hours. Both devices were attached to the waist with an elastic belt. After a short instruction, the children were asked to wear the belt from waking time to bedtime for 7 consecutive days. They were asked to remove the belt during activities where the devices might get wet (e.g. swimming, showering). The parents were asked to recharge the GPS receivers during the evening when the children were asleep. Children and parents could read back instructions in a manual that was handed out together with the devices.

Body height and weight of the children were measured with a microtoise (Stanley 04–116) and a digital scale (Seca 812, Vogel & Halke GmbH & Co) to the nearest 0.1 cm and 0.1 kg respectively. Measured weight and height were used to calculate children’s body mass index (kg/m^2^) according to which participants were categorized into normal weight, overweight and obesity according to age- and sex-specific cut-offs for children [23]. In addition, a short questionnaire was completed by the parents to provide information on the children (i.e., date of birth, sex).

### Data analysis

Accelerometer data were downloaded to a personal computer using the manufacturer’s software (Actilife v3.6.0, ActiGraph) and further processed within SPSS Version 20.0. Periods of ≥ 20 minutes of zero counts were deemed biological implausible. It was assumed that children did not wear the belt during such periods and all periods of ≥20 minutes of zero counts were excluded from the analysis [24,25]. Only participants recording more than five hours of accelerometer data (after removal of non-wear periods) on at least two weekdays were used in the analysis. This minimum wear time of five hours was chosen to minimize data loss among the relatively small sample of participants. Weekend days were not included in the analysis because only a few children (n=15) recorded activity at the schoolyard in the weekend. Based on the findings of Trost et al. [26], moderate to vigorous physical activity (MVPA) was determined using the cut-off point of >574 counts per 15 second epoch [26,27]. There was no requirement in terms of bouts of activity. Every single epoch that was recorded above this threshold contributed to time in MVPA.

The GPS data were mapped with the URBIS III [28] software package. Exact location of the school building and schoolyard were defined with the use of TOP10NL. This contains topographic data (e.g. buildings, roads, rail road tracks, terrain water) from the digital database of the Dutch national land use register [29]. With the use of the TOP10NL data, polygons were drawn around the schoolyards of the six schools.

After construction of the polygons, accelerometer and GPS data were date- and time-matched to create a measure of activity and location for each 15 second accelerometer epoch. During this process, the location of every 15 second epoch was defined as either being on the schoolyard (a), or inside the school building (b), or as outside the school environment (c). This was done by two methods: firstly by school class hours, and secondly by GPS location.

Firstly, all accelerometer epochs that fell during school classes, when children are known to be inside, where defined as *‘(b) inside the school building’*. We chose to use these school class times instead of GPS location since the GPS may not record a position, or may record a highly inaccurate position, because inside a classroom the reception of satellite signals is often obstructed [20]. The assessment of school hours used to determine whether children were inside or outside the school building, is described further below.

Secondly, all remaining accelerometer epochs were assigned as (a) or (c) based on the three 5s GPS locations per 15s accelerometer epoch. When the majority (two or three) of the GPS epochs were situated within a distance of 10 meters of the schoolyard polygon, the accelerometer epoch was defines as *‘(a) on the schoolyard’*. The buffer of 10 meters was chosen to account for the positional accuracy of the GPS receiver (e.g. due to urban canyoning) [20]. Remaining epochs were defined as *‘(c) outside the school environment’*.

**Figure 1:**
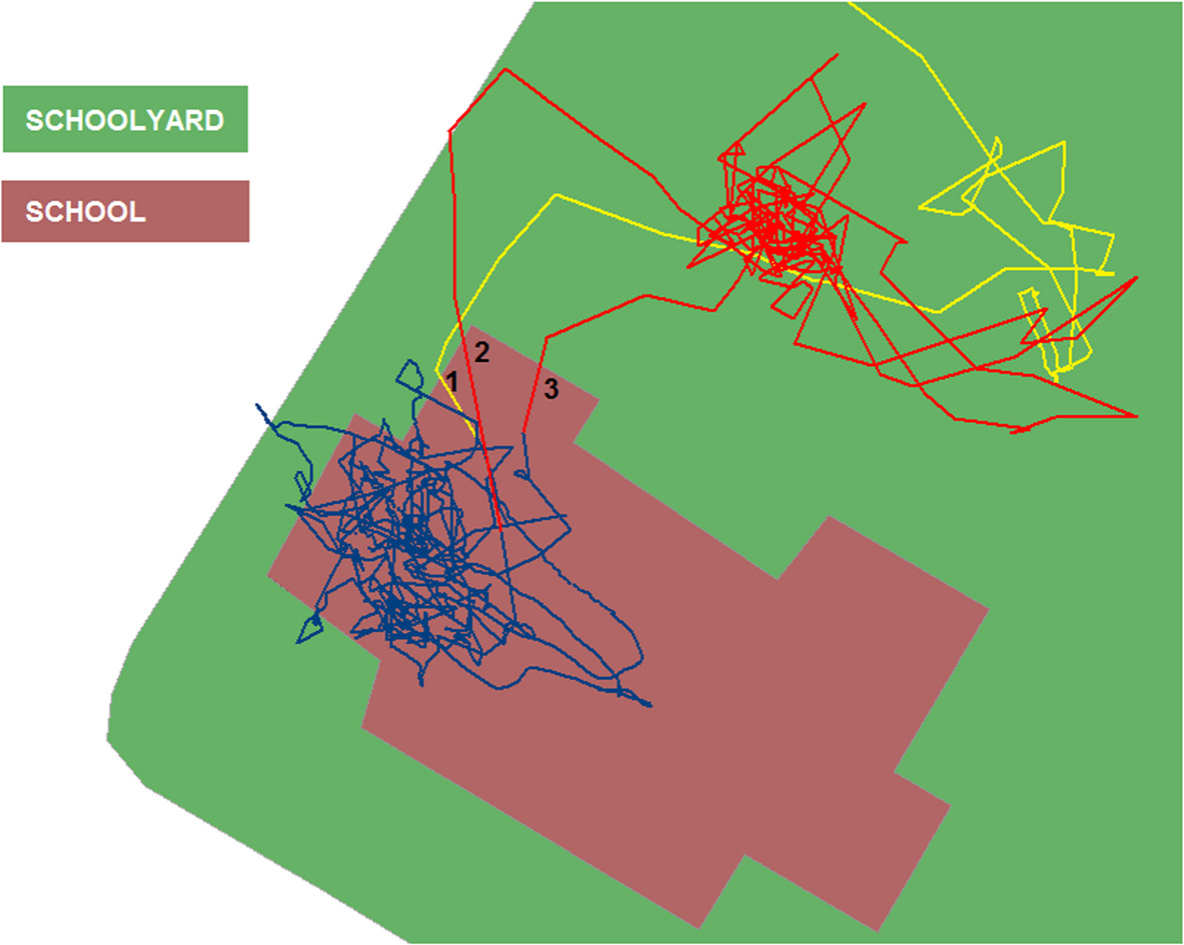
**GPS track of a child on the schoolyard and inside the school building.** Legend: Example of a child arriving at school in the morning, and entering and leaving the school building for school recess. Some of the activity recorded in the school building (blue) is projected as being on the schoolyard due to GPS inaccuracy indoors. The (yellow) pre-school segment ended when the child entered the school building (1). Likewise, the start (2) and ending (3) of the school recess segment were determined. The school recess segment is depicted as the red-colored track.

For school hours, a distinction was made between different segments of the day: *(1) pre-school, (2) school, (3) school recess, (4) lunch break,* and *(5) post-school.* The pre-school (1) segment started with children getting up from bed, the post-school (5) segment ended when the children went to bed for sleeping. The start and ending of the other segments were determined by visual exploration of the GPS-signal. This way, it was established per group of children sharing the same school program, at what time they entered or left their school building (see Figure 1). The accelerometer epochs recorded during the second segment (2) were labeled as (b) ‘inside the school building’, as described above. As can also be seen in Figure 1, some of the children’s activity inside the school was incorrectly projected as being on the schoolyard polygon because of the limited GPS accuracy indoors [20].

Furthermore, days on which children did not record data on the schoolyard were excluded from the analysis. On these days, children were considered not to have worn the GPS, or not to have visited school. After determining which accelerometer epochs were on the schoolyard, minutes of MVPA and percentage of time in MVPA were calculated for each participant and for each segment of the day. Moreover, mean physical activity per participant (mean counts per 15 seconds) was also calculated for the different segments of the day. Finally, the contribution of schoolyard MVPA to daily MVPA was determined by dividing the total number of minutes of MVPA that children recorded on the schoolyard through the total number of minutes of MVPA that children recorded during the whole day.

### Statistical analysis

Student’s t-tests were used to test differences in age, BMI, body height or body weight between the original population and the final study population. P-values < 0.05 (two-sided) were considered statistically significant. Logistic mixed-effects models were used to estimate the difference in MVPA between segments of the day. In addition, differences between boys and girls were examined as well. The statistical analysis was conducted on the accelerometer epoch level, using similar methods as Wheeler et al. [13] to take into account the clustered data structure with individual differences in wear time and the varying duration of the segments of the whole day. In this way, the relative odds of an accelerometer epoch exceeding the MVPA cut-off could be estimated for physical activity on the schoolyard. Odds ratios were calculated for all segments of the day, with pre-school as the reference category (OR= 1.00). Furthermore, differences in mean counts per 15 seconds between different segments of the day were compared with linear mixed-effects models. Analyses were performed in SPSS Version 20.0. Results for boys and girls were presented separately and models were adjusted for school attended, age and BMI category.

## Results

Initially, a group of 97 children wore accelerometers and GPS receivers. Children that did not record sufficient accelerometer data (n=13), or did not record any data on the schoolyard (n=8), were excluded from the analysis. No significant differences in age, BMI, body height or body weight were observed between the original and the final study population. Thus, the final study population consisted of 76 children, including 32 boys and 44 girls. The age of the children ranged between 6 and 11 years, with an average of 8.6 years (SD=1.40). Around one third (n=23) of the children were classified as being either overweight or obese. Further descriptive statistics are presented in Table 1. All together, the children recorded 211 days with combined GPS and accelerometer data, 12 children provided one day of data, 17 children provided 2 days, 23 provided three days and 24 provided 4 days of data. Participants wore the accelerometer for an average of 11.2 hours/day (SD±1.9). There was no significant difference in mean wear time between boys and girls. The GPS location was available for 91.3% of all accelerometer data.

**Table 1:** General characteristics of the study population

	**Total**		**Boys**		**Girls**	
	***n***	***Mean*±*SD***	***n***	***Mean*±*SD***	***n***	***Mean*±*SD***
*Age (years)*	76	8.6 ± 1.4	32	8.5 ± 1.4	44	8.6 ± 1.4
*Body height (cm)*	76	137.4 ± 9.6	32	137.8 ± 9.5	44	137.1 ± 9.9
*Body weight (kg)*	76	34.8 ± 10.5	32	34.6 ± 9.9	44	34.9 ± 11.1
	***n***	***%***	***n***	***%***	***n***	***%***
BMI Category						
*Normal*	53	69.7	24	75.0	29	65.9
*Overweight*	14	18.4	4	12.5	10	22.7
*Obese*	9	11.8	4	12.5	5	11.4
Location of school						
*Haarlem*	8	10.5	2	6.3	6	13.6
*Amersfoort*	21	27.6	11	34.4	10	22.7
*Amersfoort (2)*	9	11.8	3	9.4	6	13.6
*Hengelo*	20	26.3	10	31.3	10	22.7
*Vlaardingen*	8	10.5	4	12.5	4	9.1
*Rotterdam*	10	13.2	2	6.3	8	18.2

### Physical activity

Overall, children recorded 48.9 (SD±22.2) minutes of MVPA per day. With 56.2 (SD±23.7) minutes of MVPA per day, boys spent significantly more time in MVPA than girls (OR=1.30 [95% CI: 1.04-1.62), who accumulated 43.6 (SD±19.6) minutes of MVPA/day. Out of the 76 children, 19 children (25.0%) accumulated an average of ≥ 60 minutes MVPA a day and met the recommended amount of physical activity. This group consisted of 11 boys and 8 girls.

### Physical activity in the school environment

Table 2 provides a summary of children’s daily physical activity (in MVPA) on the schoolyard and inside the school building. When children were in the school environment, they spent most of their time inside the school building: on average this was around 4 hours/day. Only a small percentage of this time was MVPA, this was 2.1% (SD±2.1) for boys and 2.8% (SD±3.2) for girls.

**Table 2:** Children’s physical activity on the schoolyard compared to physical activity inside school

			**Schoolyard**	**Inside school**	**Whole day**
Total time (minutes) per day	Boys	*Mean*	33.3	236.5	668.4
		*(±SD)*	(±13.4)	(±48.7)	(±107.1)
	Girls	*Mean*	45.1	244.9	679.2
		*(±SD)*	(±23.9)	(±66.1)	(±124.3)
Minutes of MVPA per day	Boys	*Mean*	8.8	4.9	56.1
		*(±SD)*	(±5.1)	(±5.2)	(±23.7)
	Girls	*Mean*	7.0	7.1	43.6
		*(±SD)*	(±5.1)	(±8.2)	(±19.6)
Proportion of time spent as MVPA	Boys	*%*	27.3*	2.1	8.5*
		*(±SD)*	(±12.7)	(±2.1)	(±3.5)
	Girls	*%*	16.7*	2.8	6.5*
		*(±SD)*	(±10.4)	(±3.2)	(±2.9)

*significant difference between boys and girls (p<0.01).

Children spent an average of 40.1 (*SD*±20.9) minutes per day on the schoolyard during which they recorded 7.8 (SD = 5.1) minutes of MVPA. Of the time recorded on the schoolyard, the percentage spent as MVPA was 27.3% (SD±13.7) for boys. Girls recorded 16.7% (SD±10.4) of time on the schoolyard as MVPA. Boys recorded higher numbers of MVPA/ minute when they were on the schoolyard (OR=2.11 [95% CI 1.54-2.90]). Most of the minutes of MVPA per day were accumulated outside the school environment, 42.5 (SD±21.9) minutes for boys and 29.5 (SD±14.7) minutes for girls. Schoolyard physical activity contributed towards 17.5% and 16.8% of boys’ and girls’ total minutes of MVPA. The proportion of time spent in MVPA on the schoolyard, for boys 27.3% (SD±12.7) and girls 16.7% (SD±10.4), was therefore much higher than the overall proportion of time spent in MVPA over the day (8.5% (SD±3.5) for boys and 6.5% (SD±2.9) for girls, respectively).

### Schoolyard physical activity during the different segments of the day

**Figure 2:**
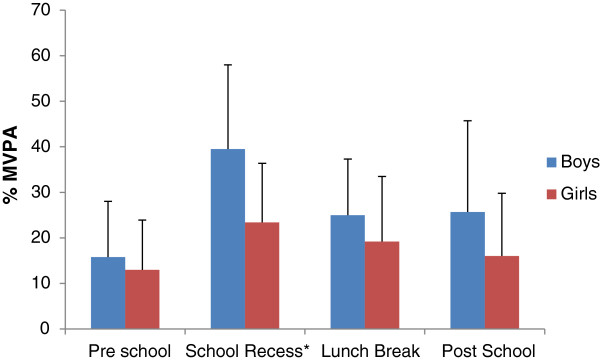
**Percentage of time on the schoolyard spent as MVPA: different segments of the day.** Legend: *Difference in odds to record MVPA between boys and girls: OR=2.55 [95% CI: 1.69 – 3.85], p<0.01.

For the different segments of day, percentage of time spent in MVPA is shown in Figure 2 (for total number of minutes spent on the schoolyard, see Table 3). Children were most intensively physically active on the schoolyard during school recess (boys: OR= 4.23 [3.62-4.95], girls: OR 2.56 [2.21-2.97], compared to the pre-school segment, see Table 4). During school recess, children recorded 18.3 (SD±7.6) minutes on the schoolyard. For boys, during school recess the percentage of MVPA was 39.5% (SD±18.5) whereas girls recorded 23.4% (SD±13.0) of the time in MVPA. Differences between both sexes were significant (OR=2.55 [95% CI: 1.69 - 3.85]). Results of the statistical analysis are presented in Table 4, with the pre-school segment as the reference category.

**Table 3:** Description of total time recorded on the schoolyard for different segments of the day

			***Pre school***	***School recess***	***Lunch break***	***Post school***	***Total***
Total minutes spent on schoolyard	Boys	Mean	6.3	15.7	12.9	11.1	33.3
		(±SD)	(±3.6)	(±6.3)	(±12.8)	(±10.6)	(±13.4)
	Girls	Mean	6.0	20.1	19.3	14.5	45.1
		(±SD)	(±3.5)	(±8.0)	(±19.5)	(±16.5)	(±23.9)

**Table 4:** Odds ratio of an epoch on the schoolyard exceeding the MVPA cut-off (>574 counts) for different segments of the day (adjusted for age, BMI category and school attended)

	**Boys (n=32)**				**Girls (n=44)**			
	***N***	***OR***	***CI***	***p***	***N***	***OR***	***CI***	***p***
Pre school	1670	1.00			2210	1.00		
School recess	3945	4.23	[3.62–4.95]	< 0.01	6582	2.56	[2.21–2.97]	< 0.01
Lunch break	2737	2.11	[1.79–2.49]	< 0.01	6634	1.44	[1.24–1.69]	< 0.01
Post school	3696	1.41	[1.19–1.67]	< 0.01	7147	.99	[0.85–1.17]	0.97

*N* number of accelerometer epochs, *n* number of children, *OR* Odds Ratio, *CI* 95% Confidence Interval.

## Discussion

This is the first study that examined the duration and the intensity of schoolyard physical activity during several school days using objective methods to assess physical activity. The aim of this study was to investigate how long and at what intensity children are physically active on the schoolyard, not only during school recess but also during other segments of the day. Children spent an average of 40.1 minutes/day on the schoolyard. While this represented only 6.1% of total registered time, this time on the schoolyard contributed towards 17.5% and 16.8% of boys’ and girls’ minutes of MVPA. Ridgers et al. [18] found very similar numbers, with time spent during school recess contributing towards around 17% of children’s total school day MVPA.

The intensity of schoolyard physical activity varied during the different segments of the day. On the schoolyard, children in this study recorded the highest intensity of physical activity during school recess. Boys spent 39.5% (SD±18.5) of the time during recess in MVPA compared to 23.4% for girls. Previous studies [18,30] have suggested a guideline of 40% of MVPA during recess periods. In this study, 43.8% (n=14) of boys and 11.4% (n=5) of girls reached this percentage during school recess on the schoolyard.

During school recess children spent a relative high percentage of time in MVPA. Thus, the results of this study seem to confirm the importance of school recess for the accrual of minutes of MVPA. They support the recommendation recently made by Ridgers et al. [18], that policy makers should ensure that all children in primary school should have at least one recess period a day to provide an opportunity for MVPA. Whether adding extra school recess time can contribute towards reaching MVPA requirements needs further investigation since current results are all based on cross-sectional data [17-19]. When considering the time at school dedicated to school recess, a relevant notion is the suggested beneficial effect of physical exercise for children’s cognitive development; a review by Trudeau and Shephard [31] suggests that this extra time for physical activity during school hours might be beneficial for academic performance.

Although in the present study proportionally more of the time on the schoolyard is spent as MVPA, it should be noted that most of the minutes of MVPA during schooldays were accumulated outside school hours and on other locations than the schoolyard. Moreover, almost no minutes were recorded on the schoolyard in the weekend or in the evening. Partially, this might be because most of the data was collected during winter when children are least active outside [32]. Also, in the current study, physical education lessons could not be differentiated from other activities. Most children had physical education outside their school building on other locations. Results from this study seem to confirm the finding by McGall et al. [19] that physical activity opportunities in the school environment alone were not enough for children to accrue adequate numbers of MVPA.

Remarkable are the low numbers of MVPA that were accumulated when children were inside school. For boys, only 2.1% (SD ±2.1) of time (see Table 2) that was recorded inside the school building was spent as MVPA. Girls recorded 2.8% (SD ±3.2) of the time inside school as MVPA. Nettlefold et al. [17] found slightly higher numbers during regular class time: 12.0% of time was spent as MVPA for girls and 14.1% for boys. This difference can partially be explained because of different threshold values used to define MVPA. The same study found that children spent 70% of regular class time as sedentary activity. A more recent study by Ridgers et al. [33] also showed that around 63% of class time is spent sitting. These number are in line with the findings of the current study that primary school children are relatively inactive when they are inside the school building. These low activity levels during class stress the importance of promoting physical activity outside class hours (e.g. at recess- and lunchtime by walking and cycling to school [34,35]), but also the importance of interrupting sedentary behavior inside the school building (e.g. in-class physical activity breaks, environmental cues and prompts such as standing easels [36]). Recent studies suggest that physical activity during the school day, may stimulate children’s attention and academic performance [34,37-39]. During almost all segments of day, boys were more physically active on the schoolyard than girls. Other recent studies, which also used accelerometry to measure physical activity, show similar results [17-19]. Thompson et al. [40] indicated that stage of maturation has an influence on the amount of physical activity, with more mature children being less physically active. Since girls mature earlier than boys, this might explain the difference observed between sexes. Moreover, Blatchford et al. [41] showed that boys and girls engage in different sorts of activities on the schoolyard. Boys are more likely to be involved in ball games and more vigorous play. Girls were most likely to engage in social conversation, sedentary play and skipping and avoid rough physical contact during play. In this respect, Ridgers et al. [18] recommend schools to consider organizing playgrounds to allow equal access to activities for boys and girls. Sallis et al. [42] have shown that improving the design of the play environment and extra equipment can be a beneficial strategies to promote girl’s physical activity levels. However, more detailed research on the effect of such interventions to reduce the observed differences between sexes is still needed.

### Limitations

Compared to self-reports, questionnaires or observations, accelerometry combined with GPS is an objective method to measure physical activity [4]. However, the use of these objective measures also has its limitations. There is a chance of logging inaccurate positions because of signal inaccuracy (e.g. due to urban canyoning) and noise, especially when children are indoors [20]. In the current study, at least three GPS locations were available for each single accelerometer epoch, thus reducing positional inaccuracy. Furthermore, children’s school hours were determined to further reduce the chance of incorrectly classifying children as being on the schoolyard. Besides these issues associated with GPS measurements, accelerometer measurements also have their pitfalls. The threshold value to determine minutes of MVPA remains arbitrary and can have a considerable impact on the percentage of time spent in MVPA [43]. Because of the issues associated with choosing the right threshold for MVPA, this study also reports the accelerometer output independent of a threshold, in mean counts per 15 seconds (Additional file 1: Table S1). When using this outcome measure, differences between the segments of the day are similar to differences in the percentage of MVPA, with school recess as the segment where children are most active. Moreover, criteria used for minimum wear time could also have affected the outcome of the accelerometer measurements. The relative low wear time of some of our participants would mainly affect the analysis of accumulated minutes of MVPA during different segments of the day. We tested whether the accumulated minutes of MVPA were different for subjects with less than 10, and more than 10 hours of accelerometer data/day. This was not statistically different when tested with a student’s *t*-test. Future studies can consider to further improve classification of children’s activity levels by using additional methods to measure physical activity, such as heart rate monitoring [44].

Furthermore, results from this study are likely to be affected by seasonal influences and may not represent average physical activity levels over the entire year. Most of the data was collected during winter (December 2008-April 2009), this is commonly the time of year when children are least active because of the fewer daylight and poorer weather conditions [32,45]. The winter season could have influenced the number of children on the schoolyard in the weekend and evening. Due to the low number of children that were present on the schoolyard during weekends or evenings in the current study, it was not possible to compare these segments with the other segments of the day.

Because of the relative small number of children that participated in each school, characteristics of the schoolyard and their association with physical activity could not be assessed. By making changes in the physical school environment (e.g. by placing playground markings on the schoolyard [46]), it seems possible to improve children’s physical activity participation with low cost interventions [47,48]. Future research using GPS and accelerometry is warranted to assess which physical and social elements of the schoolyard (e.g. surface area, available equipment, supervision, lighting) are associated with higher levels of physical activity amongst children. This may also provide more insight into the observed differences between boys and girls.

## Conclusions

The proportion of time spent in MVPA is relatively high on the schoolyard compared to the total day, and the time inside the school building. Most of the minutes of MVPA on the schoolyard occurred during school recess. The schoolyard appears to be an important setting for children’s physical activity, especially during school recess. Moreover, children showed relative sedentary behavior when they were inside the school building. Policy makers should thus realize that school recess provides an excellent opportunity to accumulate minutes of MVPA. Interventions that focus on the promotion of physical activity during the school day have the difficult challenge to activate girls, as they currently lag behind in physical activity levels during all segments of the school day.

## Competing interests

The authors declare that they have no competing interests.

## Authors’ contributions

DD carried out data analysis, interpretation of data and wrote the manuscript. FP provided overall supervision and review for the present manuscript, helped conceive the study, and was involved with data acquisition and study design. RPS organized the data for data analysis and supported with interpretation of data. PVD helped with statistical analysis, interpretation of data and approved the manuscript. JM was involved with drafting the manuscript and study design. SIV was responsible for study conception, data acquisition and revised the manuscript critically. All authors read and approved the final manuscript.

## Supplementary Material

Additional file 1: Table S1 Mean counts per 15 second epoch, inside school and on the schoolyard. Mean counts on the schoolyard are further differentiated for segment of the day. *Differences between school recess and all other segments of day are significant (p<0.01). Click here for file
