# Supplementary material for: Schoolyard physical activity of 6–11 year old children assessed by GPS and accelerometry
Source: Int J Behav Nutr Phys Act. 2013 Aug 14;10:97. doi: 10.1186/1479-5868-10-97 (PMC3751773; doi:10.1186/1479-5868-10-97)
Supplement: Additional file 1: Table S1 — Mean counts per 15 second epoch, inside school and on the schoolyard. Mean counts on the schoolyard are further differentiated for segment of the day. *Differences between school recess and all other segments of day are significant (p<0.01). [file 1479-5868-10-97-S1.pdf]

Additional file 1: Table S1. Mean counts per 15 second epoch, inside school and on the schoolyard. Mean counts on the schoolyard are further differentiated for segment of the day.

		Location	Mean	(±SD)
Counts per 15 sec.	Boys (n=32)	Inside School	59.7	(±34.0)
		Schoolyard	388.9	(±135.5)
		- Pre school	260.2	(±98.9)
		- School Recess*	523.9	(±180.3)
		- Lunch Break	377.7	(±146.8)
		- Post School	376.1	(±203.5)
	Girls (n=44)	Inside School	78.0	(±87.6)
		Schoolyard	289.5	(±132.1)
		- Pre school	238.3	(±121.5)
		- School Recess*	382.3	(±171.9)
		- Lunch Break	310.3	(±143.1)
		- Post School	274.3	(±157.3)

*Differences between school recess and all other segments of day are significant ($p<0.01$).
